# Retargeting *Clostridium difficile* Toxin B to Neuronal Cells as a Potential Vehicle for Cytosolic Delivery of Therapeutic Biomolecules to Treat Botulism

**DOI:** 10.1155/2012/760142

**Published:** 2011-09-20

**Authors:** Greice Krautz-Peterson, Yongrong Zhang, Kevin Chen, George A. Oyler, Hanping Feng, Charles B. Shoemaker

**Affiliations:** ^1^Division of Infectious Diseases, Department of Biomedical Sciences, Tufts Cummings School of Veterinary Medicine, 200 Westboro Road, North Grafton, MA 01536, USA; ^2^Synaptic Research LLC, 1448 South Rolling Road, Baltimore, MD 21227, USA

## Abstract

Botulinum neurotoxins (BoNTs) deliver a protease to neurons which can cause a flaccid paralysis called botulism. Development of botulism antidotes will require neuronal delivery of agents that inhibit or destroy the BoNT protease. Here, we investigated the potential of engineering *Clostridium difficile* toxin B (TcdB) as a neuronal delivery vehicle by testing two recombinant TcdB chimeras. For AGT-TcdB chimera, an alkyltransferase (AGT) was appended to the N-terminal glucosyltransferase (GT) of TcdB. Recombinant AGT-TcdB had alkyltransferase activity, and the chimera was nearly as toxic to Vero cells as wild-type TcdB, suggesting efficient cytosolic delivery of the AGT/GT fusion. For AGT-TcdB-BoNT/A-Hc, the receptor-binding domain (RBD) of TcdB was replaced by the equivalent RBD from BoNT/A (BoNT/A-Hc). AGT-TcdB-BoNT/A-Hc was >25-fold more toxic to neuronal cells and >25-fold less toxic to Vero cells than AGT-TcdB. Thus, TcdB can be engineered for cytosolic delivery of biomolecules and improved targeting of neuronal cells.

## 1. Introduction

Clostridial toxins in nature are remarkably efficient cell cytosol delivery vehicles with highly evolved cell-specific delivery features that may be ideal for therapeutic applications. Specifically these toxins (1) gain entry to animals; (2) survive in blood; (3) bind to target cells expressing a specific receptor; (4) penetrate the target cells; (5) deliver an enzymatically active cargo to the cytosol. *C. difficile* toxins A and B (TcdA and TcdB) contain a receptor-binding domain (RBD) that binds to receptors that are broadly expressed on cells and then enters by endocytosis. Once in the endosome, the toxins employ a translocation domain (TD) to deliver a glucosyltransferase (GT) to the cytosol which inactivates Rho GTPases and leads to cell death [1]. The toxins also contain a cysteine protease (CPD), located between GT and TD, that cleaves the GT enzymatic “cargo” from the “delivery vehicle” at the endosomal membrane and liberates it into the cytosol [2–4]. 

*C. difficile* bacteria generally reside in the gut where the released toxins intoxicate intestinal epithelial cells and cause the disruption of tight junctions of epithelium and its barrier function. It is likely that in severe cases of the infection, the toxins penetrate into the submucosa and disseminate systemically [5]. We recently identified *C. difficile* toxins in the blood of the experimentally infected animals [6, 7], suggesting that the toxins may be reasonably stable in serum. 

Recent developments have enabled the application of TcdA and TcdB as therapeutic delivery vehicles. The *Bacillus megaterium* (*B. megaterium*) expression system has been shown to permit high-level expression of functional recombinant TcdB (5–10 mg/L culture) [8]. Secondly, the toxicity of these agents is virtually eliminated by introducing two point mutations within the GT domain that should have no effect on endosomal uptake and translocation to the cytosol (Haiying Wang and Hanping Feng, unpublished data). Finally, the limits of the GT, TD, and RBD domains have recently been carefully defined in the literature [3], facilitating efforts to replace one or more domains with similar domains from other toxins. We recently showed that it was possible to replace the RBD from TcdB with the RBD from TcdA and retain most or all of the toxin activity (Haiying Wang and Hanping Feng, unpublished data). 

One drawback to the use of TcdA or TcdB as cytosolic delivery vehicles is the lack of cell specificity. This is in contrast to botulinum neurotoxins (BoNTs) which display a marked specificity for neuronal cells. BoNTs are CDC Category A biodefense threat agents that cause paralysis by entering the presynaptic terminal of motor neurons and inhibiting neurotransmitter release. All seven BoNT serotypes bind to a neuronal receptor through a receptor-binding domain. The toxins then undergo endocytosis, delivery of the BoNT protease cargo to the cytosol, and subsequent cleavage of SNARE proteins [9–11]. Reversal of neuronal intoxication must involve either the inhibition and/or elimination of the protease. We and others have reported development of biomolecules that potently inhibit BoNT protease [12–14] or promote its degradation [15, 16]. In this study, we demonstrate that biomolecules fused to the amino terminus of TcdB can be successfully delivered to the cytosol of cells and that replacement of the TcdB RBD with the equivalent RBD from BoNT serotype A (BoNT/A) leads to a chimeric toxin with enhanced specificity for neurons. These results indicate that it may be possible to develop therapeutic agents based on TcdB in which biomolecules are delivered to BoNT-intoxicated neurons that inhibit and/or destroy the toxin protease. Such a treatment would promote accelerated neuronal recovery from intoxication and thus could serve as the first antidotes for treatment of botulism.

## 2. Materials and Methods

### 2.1. Bacterial and Mammalian Cell Cultures

Bacterial cultures of* Escherichia coli *(TOP10 cells; Invitrogen, Carlsbad, CA) and *B. megaterium *(MS941 strain; kindly provided by Dr. Rebekka Biedendieck, Germany) were grown at 37°C in Luria-Bertani (LB) medium, containing ampicillin (Amp) and tetracycline (Tet), respectively. 

Mammalian cell lines were obtained from ATCC (Manassas, VA) and cultured as monolayers in 100 mm cell culture dishes at 37°C and 5% CO_2_. Cells were reseeded twice a week after harvest using 0.05% trypsin to suspend cells. The murine neuroblastoma cell line, Neuro2A, and the human neuroblastoma line, M17, were cultured in DMEM/F12 medium (Invitrogen) supplemented with 10% fetal calf serum, 2 mM L-glutamine, 100 units/mL penicillin, and 50 *μ*g/mL streptomycin sulfate. Vero cells (kidney epithelial cells from African green monkey) were cultured in DMEM (Invitrogen) supplemented with 10% fetal calf serum, 2 mM L-glutamine, 100 units/mL penicillin, and 50 ug/mL streptomycin sulfate.

### 2.2. Cloning of TcdB Constructs

**Figure 1:**
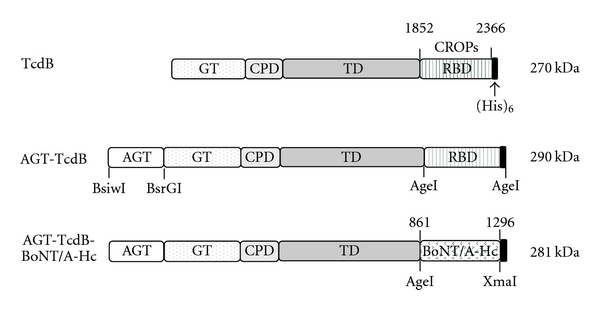
Engineered recombinant TcdB proteins. Native TcdB contains a glucosyltransferase domain (GT), a cysteine-protease domain (CPD), a translocation domain (TD), and a receptor-binding domain (RBD) as shown. The AGT-tag coding DNA was appended to the amino terminus in frame with the full-size TcdB coding DNA to create the AGT-TcdB expression vector. The TcdB RBD was replaced in frame with the full-size BoNT/A heavy chain carboxyl terminus (BoNT/A-Hc, amino acids 861–1296), containing the receptor-binding domain for BoNT/A, to generate AGT-TcdB-BoNT/A-Hc. The sizes of the boxes are approximately proportional to the sizes of the protein domains. Restriction sites used in preparing the constructions are indicated.

Full-length TcdB was expressed in *B. megaterium *as described previously [8]. To generate AGT-TcdB, the DNA encoding an alkylguanine-DNA alkyltransferase (AGT) flanked by 5′-BsiWI and 3′-BsrGI sites was synthesized (Geneart, Germany). AGT is a commercially available tag for producing AGT-fusion proteins. AGT catalyzes its own covalent binding to the substrate benzylguanine (BG) derivatives. The BG derivatives can be labeled with probes such as biotin or fluorescein to permit detection and/or cell localization of AGT-fusion proteins [17]. The AGT was appended in frame to TcdB by digestion of AGT with BsiWI and BsrGI and ligation into pHis1525/TcdB digested with BsrGI as represented in Figure 1. To generate AGT-aTcdB-ΔGT construction, a unique BamHI site (position 542) was created between coding sequences of GT and CPD by overlapping PCR. Then the AGT-tag coding DNA precisely replaced the GT in frame with the CPD, by ligation into pHis1525/TcdB digested with 5′-BsrGI and 3′-BamHI.

To generate AGT-TcdB-BoNT/A-Hc, an AgeI site was installed between the TD and RBD of TcdB. The entire RBD of TcdB, consisting of the C-terminally combined repetitive oligopeptides (CROPs, residues 1852–2366), was then replaced by the heavy chain C-terminus of BoNT/A (BoNT/A-Hc, residues 861–1296). BoNT/A-Hc coding DNA [18] was amplified by PCR from BoNT/A coding DNA and flanked by 5′-AgeI and 5′-XmaI restriction sites using primers; sense: 5′-cgaccggtggtggaggcggttcaggcggaggtggctctggcggtggcggttcccgcctgctgtcaactttcac-3′ and antisense: 5′-cggcccgggttagtgatggtgatggtgatggagaggacgttcaccccaac-3′. A flexible spacer (GGGGS)_3_ was encoded in the forward primer to separate TcdB and BoNT/A-Hc in the chimera. The reverse primer encoded a His_6_ sequence at the carboxyl coding end. All plasmid constructions were confirmed by DNA sequencing and transformed into *B. megaterium* for protein expression as described previously [8]. All DNA cloning and plasmid construction were performed at Tufts University and approved by the Institutional Biosafety Committees in agreement with NIH Recombinant DNA technology guidelines.

### 2.3. Characterization of Recombinant TcdB Chimeric Proteins

Expression and purification of His-tagged TcdB proteins was performed essentially as described previously [8] with a few modifications. 

For Western blots, AGT-TcdB and AGT-TcdB-BoNT/A-Hc were separated on a 4–20% gradient polyacrylamide gel by SDS-PAGE. AGT-tag fused to TcdB was detected using a rabbit polyclonal serum anti-AGT (New England Biolabs, Boston) at a dilution of 1 : 1000. Detection of full-length TcdB was performed using an alpaca polyclonal anti-TcdB serum, generated in our laboratory and diluted 1 : 10^6^. The BoNT/A-Hc domain in AGT-TcdB-BoNT/A-Hc chimeric protein was detected by a mouse anti-BoNT/A-Hc monoclonal antibody (A11G12.4B- kindly provided by Dr. Jean Mukherjee, Tufts University) diluted at 1 : 25,000. Detection was performed using Amersham ECL Western Blotting Detection Reagents for chemiluminescence (GE Healthcare, UK).

### 2.4. AGT-Tag Labeling with Biotin

AGT fused to TcdB was labeled *in vitro* with biotin in the absence of DTT, according to the manufacturer's instructions (New England Biolabs). Briefly, 2 *μ*M AGT-TcdB was mixed with 3 mM BG-biotin (AGT substrate labeled with biotin) in a 25 *μ*L reaction and incubated overnight at 4°C. Biotin-labeled AGT-TcdB was analyzed by Western blot using streptavidin conjugated to horseradish peroxidase (HRP). DTT was not added in the labeling reaction as recommended, because it has been reported that DTT mimics intracellular activation of the toxin [19].

### 2.5. Cytotoxicity Assay

Cell lines at semiconfluence were treated with purified TcdB, AGT-TcdB, or AGT-TcdB-BoNT/A-Hc in 5x serial dilutions starting from 100 ng/mL. Cells were cultured for a period of 24 h, and morphological changes were monitored every hour by light microscopy. Cell toxicity was quantified as the percentage of rounded cells per total cells.

## 3. Results

### 3.1. Construction and Expression of AGT-TcdB and AGT-TcdB-BoNT/A-Hc

To test the potential of TcdB-based vectors for delivery of biomolecules to the cytosol of neuronal cells, DNAs were created that encode two chimeric forms of TcdB fused to a C-terminal His_6_-tag (see diagrammatic representations in Figure 1). In the AGT-TcdB construct, an alkylguanine-DNA alkyltransferase, referred to as AGT, was appended to the TcdB N-terminus in frame with the GT coding DNA. Another construct was also prepared in which the GT was replaced by AGT, but this failed to yield meaningful amounts of full-size protein apparently because of proteolysis (data not shown) and was not further pursued. For the second chimeric toxin, AGT-TcdB-BoNT/A-Hc, the putative receptor-binding domain (RBD) from AGT-TcdB was replaced in frame with the well-defined BoNT/A receptor-binding domain [20, 21] designated BoNT/A-Hc. The recombinant TcdB chimeric proteins were expressed in *B. megaterium* and purified by nickel affinity as previously described for wild-type TcdB [8].

### 3.2. Expressed AGT-TcdB Retains AGT Enzymatic Activity

**Figure 2:**
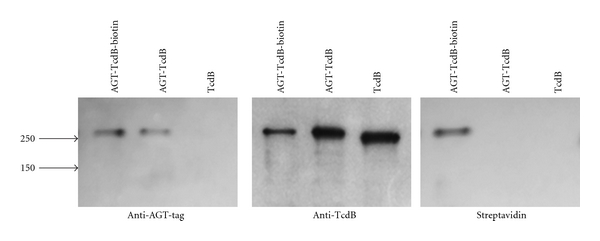
AGT-tag expressed at the N-terminus of TcdB retains enzymatic activity. Recombinant TcdB and AGT-TcdB were expressed and purified. 250 ng aliquots of purified TcdB and AGT-TcdB (± autobiotinylation) were analyzed by Western blots using anti-AGT-tag sera (left) and by anti-TcdB sera (middle). The positions of molecular weight markers are indicated with arrows. Each of the protein preparations was subjected to enzymatic reactions with BG-biotin and analyzed for bound biotin by Western blot with streptavidin (right). The data shown are representative of 2 independent experiments.

Recombinant AGT-TcdB was expressed, and the purified protein had the expected molecular weight. Western blots with polyclonal anti-TcdB serum recognized both the parental TcdB and AGT-TcdB, while AGT antiserum recognized only the AGT-TcdB (Figure 2). To confirm the proper folding and function of the AGT fusion partner, the enzymatic activity of the AGT alkyltransferase was tested. AGT-TcdB was incubated with BG-biotin which catalyzes the covalent linkage of biotin to AGT. The efficiency of *in vitro* AGT-protein labeling is generally ~95% according to the manufacturer (New England Biolabs). Western blotting with streptavidin demonstrated that AGT-TcdB became biotinylated following incubation with BG-biotin (Figure 2), thus demonstrating that the AGT domain in recombinant AGT-TcdB fusion retained enzymatic activity.

### 3.3. TcdB Delivers Glucosyltransferase Fusion Protein Cargo to the Cell Cytosol

**Figure 3:**
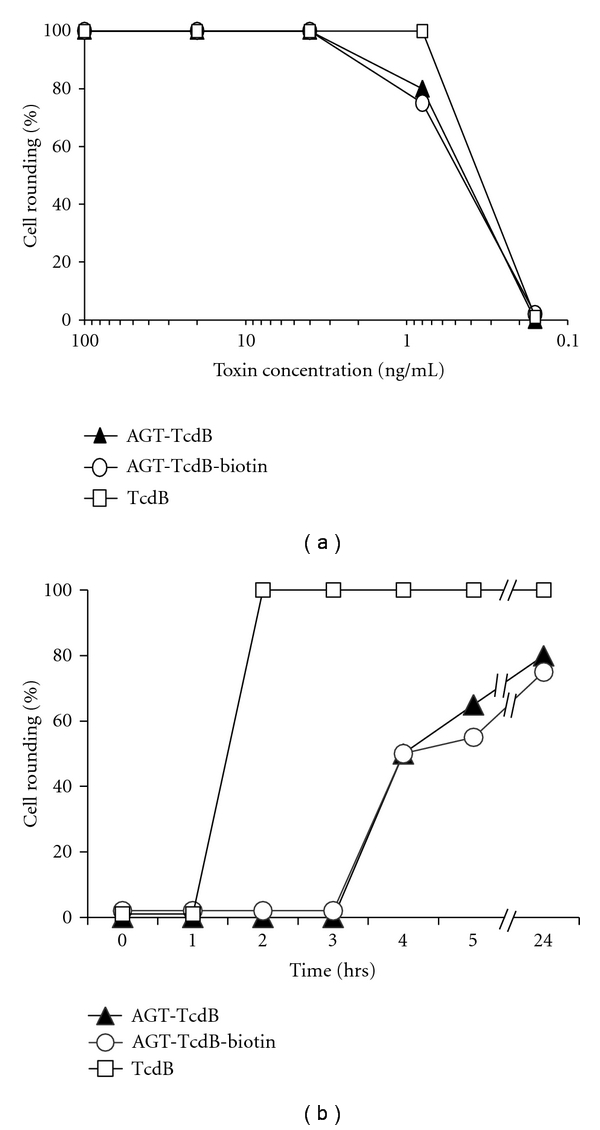
TcdB with an N-terminal AGT-tag retains cytotoxic potency. (a) AGT-TcdB potency as a function of protein concentration. Vero cells were treated with serial dilutions of TcdB and AGT-TcdB ± autobiotinylation. The dilution series started at 100 ng/mL and continued with fivefold serial dilutions. The % cell rounding was assessed after 24 hr for each concentration tested. (b) AGT-TcdB potency as a function of time after exposure. Vero cells were exposed to 0.8 ng/mL of TcdB or AGT-TcdB, and the % cell rounding was assessed hourly for five hours and then after 24 hr. The data shown in both (a) and (b) are representative of 3 independent experiments.

Cytosolic delivery of the GT domain is a requirement for cytotoxicity [22, 23]. Since each of the TcdB chimeric proteins (Figure 1) contain a fully functional GT domain, delivery of the AGT/GT fusion cargo into the cell cytosol could thus be assessed by measuring cell toxicity. In a dose-response toxicity assay in Vero cells, AGT-TcdB was found to retain a potency nearly equal to wild-type TcdB (Figure 3(a)). Cells exposed to the lowest toxic dose of AGT-TcdB were somewhat slower to become rounded than when exposed to TcdB indicating that the presence of the AGT partner may cause a small delay in toxin entry (Figure 3(b)). Even so, the half maximal effective concentration (EC_50_) is virtually identical for both toxin forms. Enzymatic labeling of AGT-TcdB with BG-biotin did not cause any significant decrease in toxin potency (Figures 3(a) and 3(b)). We conclude that the AGT-GT fusion protein with fully functional glucosyltransferase activity is delivered to the cytosol by TcdB with an efficacy nearly that of GT alone, thus demonstrating the potential of TcdB-based vectors to function as cytosolic delivery vehicles.

### 3.4. Replacing the TcdB Receptor-Binding Domain with the Equivalent Domain from BoNT/A Increases Neuronal Cell Toxicity

**Figure 4:**
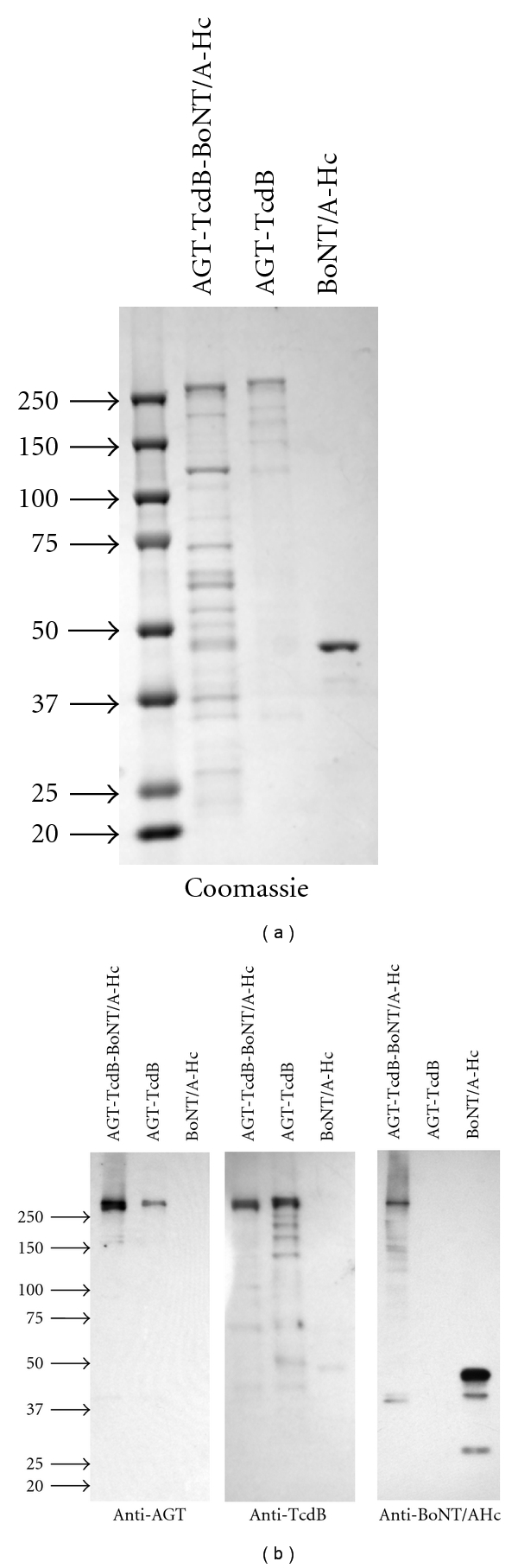
Recombinant expression of TcdB with a BoNT/A RBD. Recombinant AGT-TcdB, AGT-TcdB-BoNT/A-Hc, and BoNT/A-Hc were expressed and purified. (a) Each preparation containing 250 ng of protein was analyzed by SDS-PAGE and protein stain. (b) The preparations were also analyzed by Western blots using anti-AGT serum, anti-TcdB serum, or anti-BoNT/A-Hc mAb as indicated. The position of molecular weight markers is indicated with arrows.

Recombinant AGT-TcdB-BoNT/A-Hc protein was expressed from an expression vector in which the TcdB RBD coding region from AGT-TcdB was replaced by DNA encoding the BoNT/A RBD, the carboxyl 50 kDa portion of the BoNT/A heavy chain (Figure 1). The goal was to engineer TcdB for improved entry into neuronal cells and reduced entry into nonneuronal cells. The AGT-TcdB-BoNT/A-Hc preparation, purified only by nickel affinity, contained a protein of the predicted molecular weight for the full-size protein (~280 KDa) as well as some lower-molecular-weight species that likely result from both protein degradation and protein contamination (Figure 4(a)). Nevertheless, Western blot analysis confirmed that the 280 kDa AGT-TcdB-BoNT/A-Hc protein was full size as it stained with both anti-AGT and anti-BoNT/A-Hc antibodies (Figure 4(b)). 

**Figure 5:**
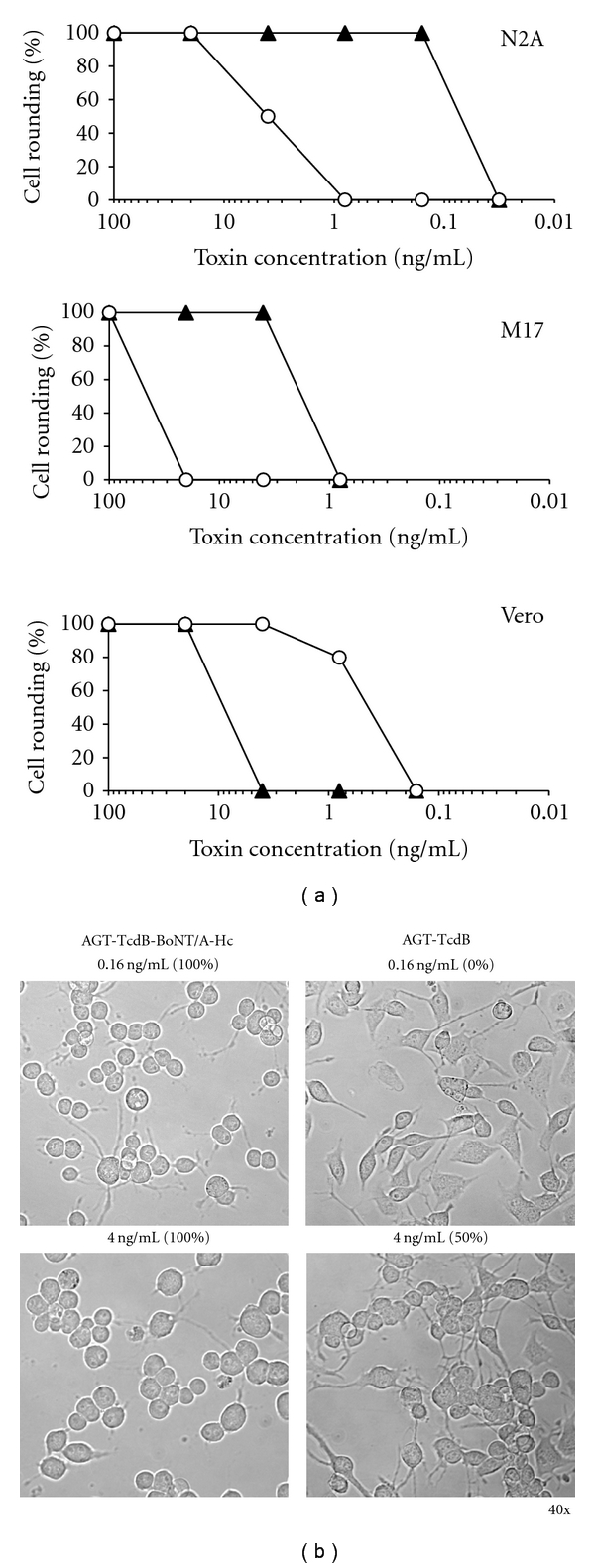
A TcdB chimera with the BoNT/A receptor-binding domain has increased specificity for neuronal cells. (a) Toxin potency of AGT-TcdB and AGT-TcdB-BoNT/A-Hc on two neuronal cell lines and Vero cells. Potency was assessed by serial dilutions of purified recombinant AGT-TcdB-BoNT/A-Hc (▲) and AGT-TcdB (∘). The potency was determined on Neuro2A and M17 neuroblastoma cells and Vero cells. Fivefold serial dilutions of the proteins were added to medium, and cells were monitored for toxicity by assessing the % cell rounding after 24 hr. Data shown are representative of 4 independent experiments. (b) Microscopic images of Neuro2A cells exposed to AGT-TcdB or AGT-TcdB-BoNT/A-Hc. Representative images are shown of Neuro2A cells exposed for 24 hr to 0.16 ng/mL or 4 ng/mL of either AGT-TcdB-BoNT/A-Hc or AGT-TcdB, respectively.

Next, we tested the cytotoxicity of AGT-TcdB-BoNT/A-Hc with AGT-TcdB on two neuronal cells lines, Neuro2A and M17, and on Vero cells, a nonneuronal cell line highly sensitive to TcdB [24, 25]. The presence of the BoNT/A-Hc domain dramatically improved the potency of the AGT-TcdB for both neuronal cell lines (Figure 5(a)). The EC_50_ of AGT-TcdB-BoNT/A-Hc was approximately 25-fold lower than AGT-TcdB when assessed 24 h following toxin exposure. In contrast, the EC_50_ of AGT-TcdB-BoNT/A-Hc for Vero cells was approximately 25-fold higher than AGT-TcdB. The finding of AGT-TcdB-BoNT/A-Hc toxicity in Vero cells (Figure 5(a)) corroborates previous reports that TcdB retains some toxicity in the absence of the putative RBD [23, 25, 26]. The rounded phenotype of the neuronal cells exposed to the two toxin forms was indistinguishable, as exemplified in representative images of Neuro2A cells (Figure 5(b)). Neuro2A cells exposed for 24 h to 0.16 ng/mL AGT-TcdB-BoNT/A-Hc were 100% rounded, while exposure to this dose of AGT-TcdB caused no observable changes compared to untreated cells. Even at 4 ng/mL, AGT-TcdB induced only 50% rounding of Neuro2A cells (Figure 5(b)).

**Figure 6:**
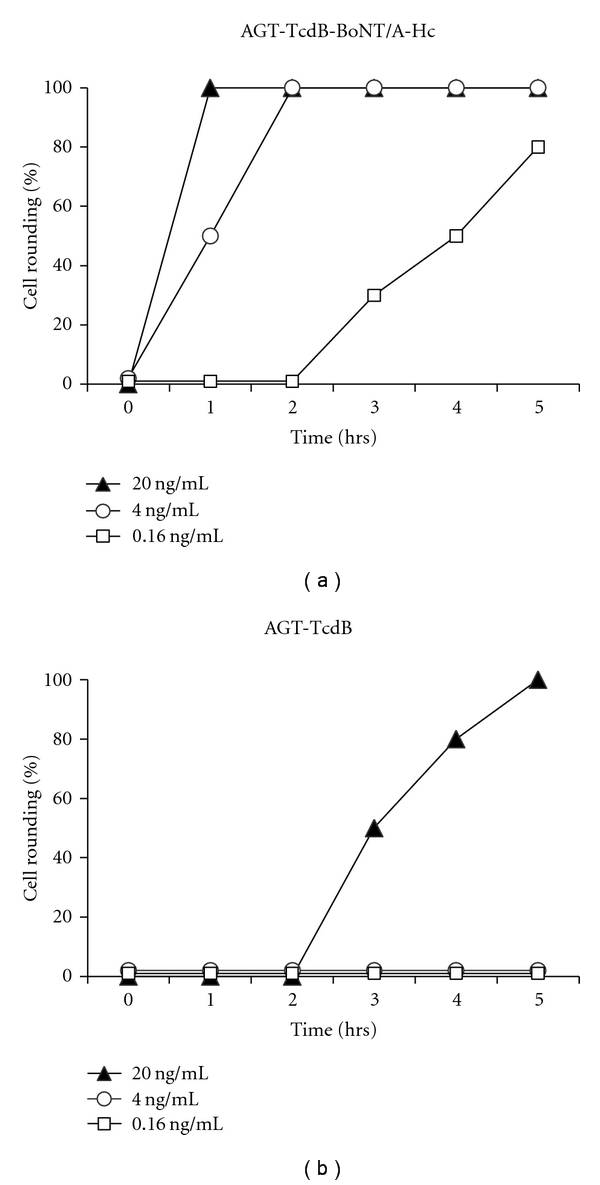
A TcdB chimera with the BoNT/A receptor-binding domain more rapidly elicits Neuro2A cell rounding. Neuro2A cells were exposed to three different concentrations (20 ng/mL, 4 ng/mL, and 0.16 ng/mL) of AGT-TcdB-BoNT/A-Hc or AGT-TcdB and the % cell rounding was assessed hourly for 5 hr. Data shown are representative of 2 independent experiments.

It is noteworthy that cell rounding was also observed to occur more rapidly in the two neuroblastoma cell lines following exposure to AGT-TcdB-BoNT/A-Hc compared with exposure to AGT-TcdB. A time course of intoxication of Neuro2A cells exposed to one of these two toxin preparations at three different concentrations clearly demonstrates that it takes about 125-fold more AGT-TcdB to achieve ≥80% cell rounding in 5 hrs than with AGT-TcdB-BoNT/A-Hc (Figure 6). These results suggest that TcdB enters and intoxicates neuronal cells significantly more rapidly and efficiently when the toxin contains the BoNT/A RBD in place of the native TcdB RBD.

## 4. Discussion

BoNT-mediated paralysis is caused by inhibition of neurotransmission in poisoned neuronal cells. This blockage is induced following delivery of the endopeptidase domain (light chain) to neurons which then inactivates one or more cytosolic proteins specifically required for neurotransmitter release. While biomolecules that inhibit or eliminate the BoNT light chain have been developed [16], the *in vivo* delivery of these therapeutic agents to the cell cytosol of intoxicated neurons to promote their recovery remains a challenge. One viable option for delivery vehicles is to reengineer clostridial toxins, which are already well evolved for delivery of their enzymatic cargo to cell cytosol, such that the toxicity is removed, while the ability to enter cells and deliver cargo remains intact. 

Here, we demonstrate that wild type TcdB can be engineered as a delivery system for selective targeting of neuronal cells. *C. difficile* toxins (TcdA and TcdB) have a major advantage over other clostridial toxins as cytosolic delivery vehicles for therapeutic biomolecules. These toxins naturally carry their own protease (cysteine-protease domain) that enzymatically cleaves and releases their cargo into the cytosol, eliminating the need to engineer a mechanism that permits this release. For example, it has been reported that the GT domain of TcdA can be removed and replaced with luciferase to generate a functional delivery vehicle capable of translocating luciferase to the cytosol of target cells [27]. Previous studies have shown that BoNTs can also be adapted as delivery vehicles, but unlike Tcds, BoNTs do not have an autocatalytic domain to release the light chain into the cell cytoplasm. Thus, in constructs in which the toxic light chain was removed, it was necessary to insert a linker that promotes a disulfide bond between the cargo and the translocation domain to make possible cargo release into the cytosol following disulfide bridge reduction in the endosome [28, 29]. Such an approach may be less efficient or ineffective for some therapeutic cargo.

In this work, we have engineered expression vectors for two chimeric TcdB proteins in which a functional GT domain remains in place. The strategy was to test the TcdB ability to deliver cargo to the cytosol by measuring the cytotoxic potency of the two chimeric proteins in comparison to wild-type TcdB. This eliminates the need for microscopic or fractionation methods to distinguish between endosomal and cytosolic cargo. In AGT-TcdB, an alkylguanine-DNA alkyltransferase, referred to as AGT, was appended to the GT domain of wild-type TcdB. Our results show that AGT was at least partially functional, since it enzymatically labeled AGT-TcdB with biotin. AGT-TcdB was capable of intoxicating Vero cells nearly as efficiently as wild type TcdB. Thus, we infer that AGT and GT domain were delivered together to the cell cytosol, and that adding a cargo to the N-terminus of the toxin did not interfere substantially with GT domain translocation and activity. Earlier work had shown that gluthatione-S-transferase (GST) could also be appended to the wild-type TcdB and detected in the cytosol of intoxicated cells, but the fusion toxin was not fully active, and there was no data as to the efficiency of delivery or that the GST fusion protein retained its enzymatic activity [22]. 

Next, we tested whether TcdB could be engineered for selective neuronal toxicity by exchanging the TcdB receptor-binding domain (RBD) on AGT-TcdB with the equivalent RBD from the neuronal-specific BoNT/A toxin, generating AGT-TcdB-BoNT/A-Hc. Our results showed clearly that neuronal cells were at least 25-fold more sensitive to the toxic effects of AGT-TcdB-BoNT/A-Hc than AGT-TcdB, and signs of intoxication appeared more rapidly. The results imply that it may be possible to engineer TcdB with specificity for almost any cell type by replacing the RBD with a RBD that binds to an appropriate receptor expressed on the target cell population. It is interesting to speculate that TcdB-BoNT/A-Hc chimeras may also be capable of transcytosis through endothelial cells in the same manner as native BoNT can accomplish because the BoNT/A-Hc domain has been shown to be sufficient for retention of this BoNT function [30]. This feature may permit delivery of therapeutic agents via oral or respiratory routes.

Although AGT-TcdB-BoNT/A-Hc showed enhanced toxicity for neuronal cells, this chimera retained some toxicity for nonneuronal cells. Previous work has also reported evidence that TcdB retains some toxicity in the absence of the putative RBD and suggested that TcdB internalization into host cells may be mediated by additional receptor-binding regions that are outside of the CROP domain of the toxin [25, 26]. Indeed, a recent report [23] has shown that deletion of the C-terminal region (residues 1500–1851) of putative translocation domain (TD) reduces cell toxicity without greatly affecting GT translocation and function. The authors conclude that this portion of the putative TD likely contains additional RBD properties. The results thus indicate that deletion or replacement of residues contributing to receptor binding within the C-terminus of TcdB putative TD will likely reduce or eliminate non-neuronal cell intoxication by TcdB-BoNT/A-Hc without loss of its capability to deliver cargo to neuronal cells.

In addition to engineering TcdB with AGT fused to the amino terminus of the GT domain, we also prepared a TcdB protein in which AGT replaced the GT domain. Expression of this fusion protein apparently produced an unstable fusion protein, resulting in very low yields of purified full-size protein. One possibility is that the removal of GT resulted in an altered conformation of the protein that resulted in activation of the cysteine protease domain which then led to autoproteolysis. The results suggest it may be important to retain the GT domain in order to maintain stability of the chimeric protein. In this case, it will be necessary to eliminate the GT enzymatic activity to render the glucosyltransferase inactive, so that the therapeutic delivery agent does not retain toxicity. Towards this goal, we recently produced an atoxic and safe TcdB, by introducing two point mutations in the GT domain (Haiying Wang and Hanping Feng, unpublished data). We expect that TcdB-based vehicles with its cargo fused to the atoxic GT domain will have better stability and perhaps lead to a product having more native conformation, thus resulting in a more efficient endosomal uptake and cargo translocation to the cytosol than TcdB vehicles lacking the GT domain. Thus, in future studies, we are opting to append foreign coding DNA to a mutated TcdB-BoNT/A-Hc rather than to replace the GT domain.

In summary, this study strongly suggests it will be possible to engineer TcdB as a cytosolic delivery vehicle of therapeutic cargo to neuronal cells, and perhaps other cell types, by redirecting its cellular binding specificities. As an example, we recently developed a small biomolecule that specifically inhibits BoNT/A protease and promotes its rapid degradation [16], and a mutated TcdB containing the BoNT/A-Hc may permit specific delivery of this therapeutic cargo to neurons as an antidote for botulism.
